# Manipulation of Gut Microbiota Influences Immune Responses, Axon Preservation, and Motor Disability in a Model of Progressive Multiple Sclerosis

**DOI:** 10.3389/fimmu.2019.01374

**Published:** 2019-06-14

**Authors:** Leyre Mestre, Francisco Javier Carrillo-Salinas, Miriam Mecha, Ana Feliú, Carmen Espejo, José Carlos Álvarez-Cermeño, Luisa María Villar, Carmen Guaza

**Affiliations:** ^1^Neuroimmunology Group, Functional and Systems Neurobiology Department, Instituto Cajal, CSIC, Madrid, Spain; ^2^Red Española de Esclerosis Múltiple (REEM), Barcelona, Spain; ^3^Servei de Neurología-Neuroimmunología, Centre d'Esclerosi Múltiple de Catalunya, Vall d'Hebron Institut de Recerca, Hospital Universitari Vall d'Hebron, Barcelona, Spain; ^4^Universitat Autònoma de Barcelona, Bellaterra, Spain; ^5^Immunology Department, Hospital Universitario Ramón y Cajal de Investigación Sanitaria (IRYCIS), Madrid, Spain

**Keywords:** Theiler's virus model, gut microbiota, Treg and Breg cells, neuroinflammation, multiple sclerosis

## Abstract

Gut microbiota dysbiosis has been implicated in MS and other immune diseases, although it remains unclear how manipulating the gut microbiota may affect the disease course. Using a well-established model of progressive MS triggered by intracranial infection with Theiler's murine encephalomyelitis virus (TMEV), we sought to determine whether dysbiosis induced by oral antibiotics (ABX) administered on pre-symptomatic and symptomatic phases of the disease influences its course. We also addressed the effects of microbiota recolonization after ABX withdrawn in the presence or absence of probiotics. Central and peripheral immunity, plasma acetate and butyrate levels, axon damage and motor disability were evaluated. The cocktail of ABX prevented motor dysfunction and limited axon damage in mice, which had fewer CD4^+^ and CD8^+^ T cells in the CNS, while gut microbiota recolonization worsened motor function and axonal integrity. The underlying mechanisms of ABX protective effects seem to involve CD4^+^CD39^+^ T cells and CD5^+^CD1d^+^ B cells into the CNS. In addition, microglia adopted a round amoeboid morphology associated to an anti-inflammatory gene profile in the spinal cord of TMEV mice administered ABX. The immune changes in the spleen and mesenteric lymph nodes were modest, yet ABX treatment of mice limited IL-17 production *ex vivo*. Collectively, our results provide evidence of the functional relevance of gut microbiota manipulation on the neurodegenerative state and disease severity in a model of progressive MS and reinforce the role of gut microbiota as target for MS treatment.

## Introduction

Multiple sclerosis (MS) is a chronic demyelinating and inflammatory neurodegenerative disease of the central nervous system (CNS), and one of the most prevalent neurological diseases. The precise etiology of MS still remains unclear, yet it is largely accepted that both the onset and progression of the disease reflects a complex interaction of genetic and environmental factors (1–4). As such, MS could be considered a neurological disease that provokes an autoimmune inflammatory reaction or an autoimmune disease that induces an attack on the myelin sheath. MS could be initiated within the CNS as a neurodegenerative process or it might even be triggered by an external agent, for example through viral infection (5, 6). Particularly, in susceptible strain of mice the intracranial inoculation of TMEV induces a biphasic disease characterized by an early acute disease associated with replication of the virus in the CNS gray matter that reach the peak at day 10 post infection. TMEV initially infects neurons during the first 2 weeks spreading thereafter through axonal transport. The susceptible strains of mice fail to complete clear the virus that mainly persist in monocytes/macrophages and glial cells albeit viral titers are scarce at the chronic stages. After a latency period, depending on viral strain, the mice develop a late chronic demyelinating disease with extensive demyelinating lesions, axonal damage, and mononuclear infiltrates in the spinal cord that resembles the pathological and clinical features of the progressive forms of MS (7). Autoimmunity in TMEV-induced demyelinating disease (TMEV-IDD) is first detected as a delayed type hypersensitivity response to the myelin proteolipid (PLP), PLP_139−151_ peptide around 60 days after the infection (8, 9). A chronic autoimmune disease is subsequently established, as epitope spreading induces autoimmune responses against other myelin peptides. The events triggering autoimmunity in MS are still not fully understood, yet it seems that genetic predisposition in conjunction with environment factors, including viral infection, could drive the onset and progress of the disease. As such, the Theiler's virus model brings together these genetic and environmental components of the disease in the context of virus-induced autoimmunity (10).

In recent years, the incidence of MS in developed countries has risen hugely. One hypothesis to explain this increase in the incidence of MS is the modification of the gut microbiota as a result of changes in diet, combined with the widespread use of antibiotics (11). Indeed, there is evidence that dysbiosis of the gut microbiota is implicated in MS (12–14), as well as in other immune related diseases like rheumatoid arthritis, type 1 diabetes or inflammatory bowel disease (15, 16). However, a functional link between the gut bacteria and MS still remains to be established. In terms of experimental models of MS, the severity of EAE (experimental autoimmune encephalomyelitis) was reduced by oral antibiotic administration (17–19). Moreover, in a spontaneous relapsing–remitting model of EAE, germ-free (GF) transgenic SJL/J mice were protected against disease, while gut colonization by commensal microbiota restored disease susceptibility (12). Likewise, mono-colonization of the gut of C57BL/6 mice with segmented-filamentous bacteria promoted Th17 accumulation in the spinal cord and restored EAE development (20). By contrast, the colonization of the same mice with *Bacteroides fragilis* and its polysaccharide A dampened symptomatology in the EAE model (21). Oral administration of *Lactobacillus spp*. and *Bifidobacterium bifidus* reduced the clinical score, increasing Treg cell number in EAE mice (22). The possibility that components of the gut microbiota may favor CNS autoimmunity was also recently suggested when microbiota transplants from twins with MS augmented the incidence of spontaneous EAE (23). Complementary studies show that gut microbiota transplants from MS patients to GF mice exacerbated the development of EAE following myelin oligodendrocyte glycoprotein (MOG) immunization (24).

Early researches of RR-MS (Relapsing-Remitting MS) patients found reductions in *Firmicutes, Proteobacteria*, and *Bacteroidetes* (25), and more recently, a study investigated 60 RRMS patients, 28 untreated patients and 43 healthy controls showing increased amounts of *Methanobrevibacter* and *Akkermansia* and reduced amounts of *Butyricimonas* in untreated patients (14). Because all the studies reporting potential dysbiosis in MS focus on RR-MS, further studies are necessary to determine whether dysbiosis occurs in all forms of MS. Hence, it is relevant to investigate gut dysbiosis in a well-established model of progressive MS, such as that triggered by intracranial TMEV infection. In an attempt to explore the effects of altered gut microbiota in the progressive forms of MS, we studied the effect of gut dysbiosis produced by oral antibiotic administration (an antibiotic cocktail—ABX) on pre-symptomatic and symptomatic stages of TMEV-IDD. We also assessed the effects of microbiota recolonization after ABX withdrawn in the presence or absence of probiotics. CNS and peripheral (spleen and mesenteric lymph nodes, MLNs) immune responses, plasma acetate and butyrate levels, microglial activity, spinal cord cytokine gene expression, axon damage, and motor disability were evaluated. We provide evidence of the functional relevance of gut microbiota manipulation on the neurodegenerative state and disease severity in mice subjected to TMEV-IDD.

## Materials and Methods

### Animals and TMEV Infection

TMEV-IDD susceptible female SJL/J mice (Harlan, Barcelona, Spain) were maintained at the Instituto Cajal (CSIC, Madrid, Spain) under conventional controlled conditions: 12 h light/dark cycle, temperature 20°C (±2°C), 40–50% relative humidity, and with *ad libitum* access to food and water. The right brain hemisphere of 4-week-old mice was inoculated with 2 ×10^6^ plaque forming units (pfu) of the Daniel (DA) strain of TMEV, diluted in 30 μL of DMEM supplemented with 10% fetal calf serum (FCS) (26). TMEV was purified in the Dr. Rodríguez's lab (Department of Neurology, Mayo Clinic) (27). Sham control mice received 30 μL of the vehicle alone. Each experimental group were housed in different cages and in different isolated racks in the same room, never mixing mice exposed to different experimental conditions. Each cage contains 5 mice subjected to the same treatment, receiving the same food and sterile water, and manipulated by the same person. Therefore, the microbial environment was the same for each mouse, although coprophagy may lead to a more homogeneous microbiota among animals in the same cage that are subjected to the same experimental conditions, but not in mice housed in different cages. All experiments were performed in strict accordance with EU (Directive 2010/63/EU) and Spanish regulations (Royal Decree 53/2013 BOE n° 34 and “Comunidad de Madrid” decree: ES 280790000184). The Ethics Committee on Animal Experimentation at the Instituto Cajal (CSIC) also approved all the procedures employed in this study (protocol number: 2013/03 CEEA-IC).

### Treatments

At pre-symptomatic stages, 55 days after virus infection, mice were provided autoclaved drinking water supplemented with the ABX cocktail over 15 days: ampicillin, 1 g/L [Sigma-Aldrich, Madrid, Spain]; metronidazole, 1 g/L [Sigma-Aldrich, Madrid, Spain]; neomycin sulfate, 1 g/L [Gibco-Invitrogen, Barcelona, Spain]; and vancomycin, 0.5 g/L [Sigma-Aldrich Madrid, Spain]. This time course is consistent with standard antibiotic administration used in other studies to alter the microbiota (28, 29). On day 70 post infection (pi—symptomatic phase), the mice treated with ABX were divided into three groups: (i) a group that continued to receive ABX until the end of the experiment (day 85 pi: TAA group, *n* = 10); (ii) a group that stopped receiving ABX and that received autoclaved water (TA, *n* = 5); and, (iii) a group that stopped receiving ABX but that were administered 3 ×10^8^ cfu (colony forming units, 100 μl) of a commercial probiotic mix, Vivomixx (see below), administered by oral gavage three times a week (TAVx, *n* = 5). Vivomixx is a multispecies probiotic composed of: *Lactobacillus paracasei* DSM 24734, *Lactobacillus plantarum* DSM 24730, *Lactobacillus acidophilus* DSM 24735, *Lactobacillus* delbrueckii *subspecies bulgaricus* DSM 24734, *Bifidobacterium longum* DSM 24736, *Bifidobacterium infantis* DSM 24737, *Bifidobacterium breve* 24732, and *Streptococcus thermophilus* DSM 24731. The corresponding control groups used in these experiments were: Sham or TMEV mice that received the vehicle alone throughout the experiment (S group or T group, *n* = 10); and Sham mice administered ABX (SA group, *n* = 5). The number of mice described above corresponds to two independent experiments.

### Evaluation of Motor Function

The development and progression of the demyelinating disease was followed in the mice by evaluating spontaneous motor function. Locomotor activity was screened on day 70 and 85 pi in an activity cage (Activity Monitor System Omnitech Electronics Inc., Columbus, OH, USA) coupled to a Digiscan Analyser, evaluating spontaneous motor activity. Horizontal (HACTV) and vertical (VACTV) activity was evaluated through the total number of beam interruptions at the horizontal and vertical sensor over a 10 min session. Motor coordination was assessed in a rotarod test, the apparatus (Ugo Basile, Milan, Italy) consisting of a suspended rod rotating at a constant or accelerating speed. Three days after training for the mice to habituate to the test, animals were subjected to a trial test for a maximum of 5 min.

### Sample Collection

Fresh fecal samples were collected from each mouse on day 70 and 85 pi and stored at −80°C. The mice were anesthetized with pentobarbital (Dolethal, 50 mg/kg body weight) on day 85 pi, and the spleen was removed, placed in cold RPMI and processed for flow cytometry. MLNs were also removed and processed for T CD4 studies *ex-vivo*. Blood samples were collected in EDTA-treated BD vacutainers to obtain the plasma, which were kept cold on ice. Anesthetized mice were perfused transcardially with saline (0.9% NaCl), and the brain and spinal cord were removed and processed as we explain in the following sections.

### Metabolite Analysis

Blood samples were centrifuged for 10 min at 1,500 g in a refrigerated centrifuge within 2 h of extraction, and 0.1 ml aliquots were stored at −20°C. The protein in the samples was precipitated with an equal volume of acetonitrile (ACN—ACN: H_2_O, 1:1) and after a 10 min centrifugation at 4°C, derivatization was carried out by mixing 40 μL of samples with 20 μL of 3-NPH and 20 μL of 120 mM EDC in 6% pyridine, and incubating for 30 min and 40°C. The samples were then diluted in 920 μL of 10% ACN and finally, the samples were filtered through a 0.22 μm PTFE filters and analyzed by mass spectrometry (LC-ESI-QQQ 8030 Shimadzu). A mixture of standards was prepared in ACN: H_2_O (1:1), with 500 mg/L of acetic, and butyric acid (Sigma-Aldrich, Madrid, Spain), and derivatization was carried out as indicated above. The injection volume was 10 μL and the gradient mode analytical conditions were: 20% phase B for 2 min, to 40% phase B to 7 min; from 40 to 100% phase B till 7.5 min; return to the initial conditions from 8 to 9 min. Phase A H_2_O + 0.01% FA; Phase B ACN + 0.01% FA; flow rate 0.6 mL/min; and run time 10 min.

### CD4 T Cells *ex vivo* Experiments

CD4^+^ T cells were isolated from the mesenteric lymph nodes (MLN) using a commercial cell isolation kit and following the manufacturer's instructions (Miltenyi, Biotec Inc.; San Diego, CA). Cells from each animal were plated (10^6^ cells/well), cultured and polarized to Th17 cells in RPMI supplemented with 2-β Mercaptoethanol (50 μM), immobilized hamster anti-mouse CD3ε (500 μg/ml: BD biosciences), hamster anti-mouse CD28 (2 μg/ml: BD biosciences, San Diego, CA, USA), rat anti-mouse IFN-γ (10 μg/ml: BD biosciences, San Diego, CA, USA), rat anti-mouse IL-4 (10 μg/ml: BD biosciences, San Diego, CA, USA), hTGF-β (5 ng/ml: Peprotech, Inc, UK), recombinant murine IL-6 (20 ng/ml: Peprotech, Inc, UK) recombinant murine IL-23 (BioLegend, CA, USA, 5 ng/ml). Three days later the cells were stimulated for 5 h with PMA (100 ng/ml: Tocris Bioscience, Bristol, UK) and ionomycin (free acid, 500 ng/ml: Tocris Bioscience, Bristol, UK), and the supernatants were collected and stored at −20°C for cytokine measurement.

### ELISA

IL-17 production by Th17 polarized cells purified from MLN was measured by solid phase sandwich ELISA using Quantikine kits (R&D systems Inc, MN, USA), according to the manufacturer's instructions: reference number M1700 for IL-17 detection. The assay's sensitivity was 5 pg/ml for IL-17.

### Tissue Processing and Immunohistochemistry

The spinal cord was fixed overnight in 4% paraformaldehyde (PFA) prepared in 0.1 M phosphate buffer (PB), cryoprotected in a 30% solution of sucrose in 0.1 M PB and frozen at −80°C. Free-floating transversal cryostat sections (30 μm thick) of the spinal cord were rinsed in PB, permeabilized in 0.1 M PB containing 0.1% Triton X-100 (PBT) and blocked for 1 h at room temperature in blocking buffer (PBT plus 5% normal goat serum—NGS). Microglia were labeled with a primary antibody against Iba-1 (ionized calcium binding adaptor molecule 1, 1:1,000: Wako Chemical Pure Industry, Osaka, Japan), while axon integrity was assessed by staining with Neurofilament-H (NF-H; 1:1,000: Millipore, Temecula, CA, USA). After washing, the sections were incubated with an Alexa Fluor-594 conjugated goat anti-rabbit antibody (1:1,000: Molecular Probes Inc, Eugene, OR, USA), washed and mounted with Mowiol. In all cases, the specificity of staining was confirmed by omitting the primary antibody. Five spinal cord sections were obtained per animal and at least three mice per group were used to quantify the area immunostained by Iba-1 or NF-H using ImageJ software (NIH, Bethesda, MD, USA). White matter area from ventral and lateral horns were analyzed by immunofluorescence on a Leica TC SP5 confocal microscope. Representative images of the surface of single microglial cell were obtained with Imaris software.

### RNA Extraction, Reverse Transcription, and RT-PCR

After removal, the cervical spinal cord was frozen immediately and kept at −80°C. Given the high content of lipids found in the spinal cord, total RNA was extracted using the RNeasy Lipid RNA extraction kit (Qiagen, Manchester, UK). Genomic DNA contamination was avoided by DNAse I (Qiagen) treatment and the RNA yield was determined with a Nanodrop^®^ spectrophotometer (Nanodrop Technologies, Wilmington, DE, USA). Total RNA (1 μg) was reverse transcribed into cDNA using poly-dT primers and a reverse transcription kit (Promega Biotech Ibérica S.L., Madrid, Spain). Real-time PCR (RT-PCR) was performed with SYBR^®^ and the oligonucleotide primer sequences were as follows: *mIL-1*β, forward 5′-TGG TGT GTG ACG TTC CCA TT-3′, reverse 5′-TCC ATT GAG GTG GAG AGC TTT C-3′; *mTNF-*α, forward 5′-AGA GGC ACT CCC CCA AAA GA-3′, reverse 5′-CGA TCA CCC CGA AGT TCA GT-3′; *mIL-4*, forward, 5′-CCACGGATGCGACAAAAATC-3′, reverse 5′-GACGTTTGGCACATCCATCTC-3′; *mIL-10*, forward 5′-TGA ATT CCC TGG GTG AGA AGC TGA-3′, reverse 5′-TGG CCT TGT AGA CAC CTT GGT CTT-3′; *mIL-6*, forward 5'-TCC AGA AAC CGC TAT GAA GTT C-3′, reverse 5′-CAC CAG CAT CAG TCC CAA GA-3′; *mRps29* forward 5'-GCC GCG TCT GCT CCA A-3', reverse 5′-ACA TGT TCA GCC CGT ATT TGC-3′. PCRs were initiated by incubation at 50°C for 2 min and 95°C for 10 min, and PCR amplification was performed over 40 cycles at 95°C for 15 s and 60°C for 1 min. The cDNA samples were analyzed in triplicate on an Applied Biosystems PRISM 7500 Sequence detection system, normalizing the mRNA expression to the *Rps29* gene in each sample and quantifying gene expression by the 2^−ΔΔCt^ method. The results are expressed relative to the sham mice for each time point.

### Flow Cytometry

A CNS or spleen leukocyte suspension was obtained as described previously (30). Freshly isolated cells (10^6^) were incubated with a Fc block (anti-mouse CD16/CD32: eBioscience, San Diego, CA, USA) and labeled with anti-mouse antibodies: PE anti-CD25; PE anti-CD3; PerCP-Cy5.5-conjugated anti-CD45; PerCP-Cy5.5-conjugated anti-B220; PE-Cy7-conjugated anti-CD39; PE-Cy7-conjugated anti-CD8a; PE-Cy7-conjugated anti-CD19; APC anti-CD3; APC-Cy7-conjugated anti-CD4; APC-Cy7-conjugated anti-CD11b (all from BD Pharmingen, San Diego, CA, USA); APC anti-CD3; and PE-Cy7-conjugated anti-CD39 (both from eBioscience, San Diego, CA, USA). For Foxp3 detection the cells were suspended in fixation/permeabilization buffer for 30 min and stained with a PE anti-Foxp3 antibody (BD Pharmingen, San Diego, CA, USA). Cell viability was assessed with the LIVE/DEAD Fixable Green Dead Cell Stain Kit or LIVE/DEAD Fixable Red Dead Cell Stain Kit (Life Technologies Corporation, OR, USA). At least 10,000 events were acquired in each experiment on a FACSaria flow cytometer (BD Biosciences, San Diego, CA, USA) and the data were analyzed using FlowJo software (v.10; Tree Star, Ashland, OR, USA).

### 16S rRNA Microbial Community Analysis

Fecal DNA was isolated using QIAamp DNA Stool Mini Kit (Qiagen; cat. no. 51504) following the stool pathogen detection protocol. DNA was extracted following the manufacturer's instructions with the subsequent modification: heating step in ASL buffer at 95°C and eluting the DNA in 50 μl AE buffer. Regions V3 and V4 of the bacterial 16S rRNA gene were amplified using as primer forward CCTACGGGNGGCWGCAG and reverse GACTACHVGGGTATCTAATCC. PCRs were initiated with the incubation at 98°C for 30 s, and the PCR amplification was performed over 20 cycles of 98°C for 10 s and 50°C for 20 s and 72°C for 20 s. Additional elongation step at 72°C for 20 s were performed. PCR conditions were changed to 28 cycles of amplification and 57°C of TM for the following groups (Sham+ABX, 70 dpi; TMEV+ABX, 70 dpi; SA, 85 dpi; TAA, 85 dpi.

Quality analyses of the reads were performed using FastQC software (http://www.bioinformatics.babraham.ac.uk/projects/fastqc/). The microbiome analysis of the paired-end reads was performed using the QIIME2 v2018.8 (Qualitative Insights into Microbial Ecology) software (31). The sequences were grouped in “sequence variants” (SVs), equivalent of 100% Operational Taxonomic Units (OTUs). Representative sequences from each cluster were queried against the Greengenes database (version 13_8) to obtain the taxonomic assignments at a 97% sequence identity using BLAST. These representative sequences were further used for phylogenetic analysis and diversity metric calculations. Sequences classified within the *Streptophyta* clade, most likely coming from chloroplast DNA in murine feed (32), were extracted for further metagenomic analysis.

### Metagenome Functional Prediction

Metagenomic predictions were made using PICRUSt (Phylogenetic Investigation of Communities by Reconstruction of Unobserved States) software (33). PICRUSt uses the normalized OTU abundance table and a predicted functional trait abundance to produce a table of functions by sample assuming, by default, that KEGG (Kyoto Encyclopedia of Genes and Genomes) Orthologs predictions are sought.

### Statistical Analysis

A principal component analysis (PCA) and an extended error bar analysis with a 95% confidence interval (CI) were performed using Statistical Analysis of Metagenomic Profiles (STAMP) software (34). The rest of the data are expressed as the mean ± SEM and they were analyzed using GraphPath Prism5 (GrapPad Software; RRID: SCR_002798). One-Way ANOVA followed by the Bonferroni *post-hoc* test were used to determine the statistical significance. In the case of non-parametric analyses, a Kruskal-Wallis Mann-Whitney U-test was applied.

## Results

### Dysbiosis by Oral Antibiotics Improve Motor Function of TMEV Mice

**Figure 1:**
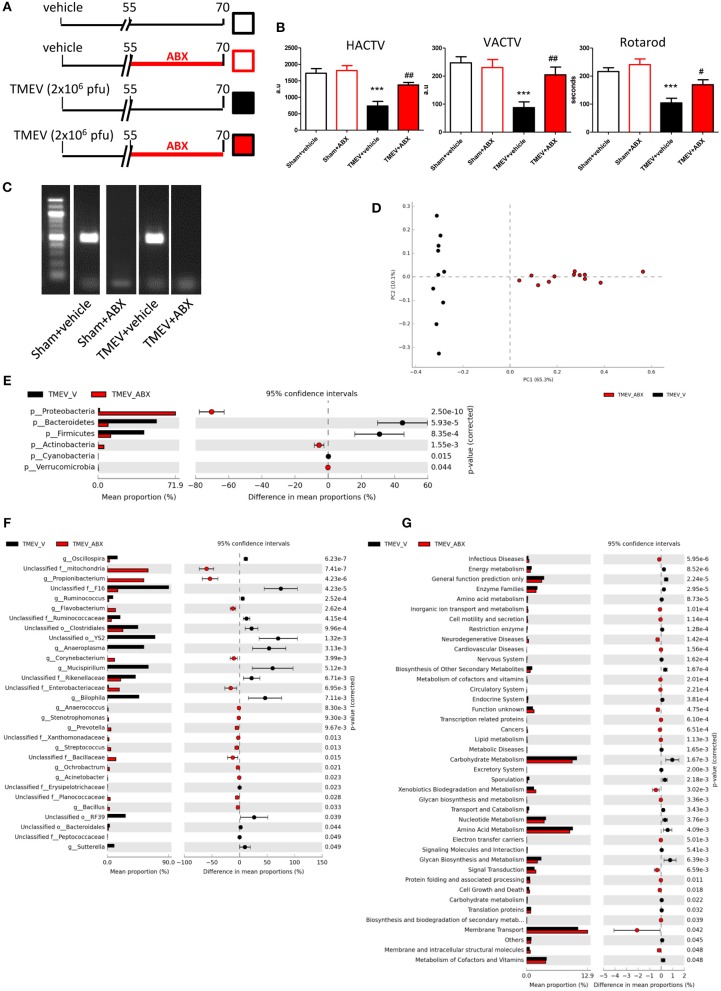
ABX-treatment prevented motor dysfunction in TMEV mice. **(A)** Diagram of the experimental groups. Mice were subjected to oral ABX treatment from day 55 to 70 pi or they received the vehicle alone. **(B)** Horizontal (HACTV) and vertical motor activity (VACTV) was evaluated for 10 min in the Activity cage, while motor coordination was measured over 5 min on a Rotarod apparatus. The data are presented as the means ± SEM: ***p < 0.001 vs. Sham + vehicle; ^#^*p* < 0.05 vs. TMEV + vehicle; ^*##*^*p* < 0.01 vs. TMEV + vehicle. **(C)** Agarose gel (1.5%) to visualize the amplicon amplified by 16S (V3–V4) PCR. **(D)** A principal components analysis of the faecal microbiota from TMEV-vehicle mice (black dots; *n* = 10) and TMEV mice treated with ABX (red dots; *n* = 12). Extended error bar plots, at phylum **(E)** and genus level **(F)**, shows the significant differences in the mean proportions of bacterial taxa in TMEV (black) and TMEV-ABX (red) mice. Corrected *p*-values are shown on the right. **(G)** Extended error bars show significantly different KEGG pathway maps in the TMEV-vehicle and TMEV-ABX mice.

Intracranial infection of a susceptible strain of mice with Theiler's virus produces motor disability that arises ~60–70 dpi (35–37) and moreover, such infection is associated with changes in the gut microbiota (30). Here we assessed whether the ABX cocktail administered orally during the pre-symptomatic phase affected the onset of the motor effects (Figure 1A). Behavior in the activity cage and rotarod performance indicated that oral ABX administration for 15 days before the appearance of symptoms apparently prevented the motor dysfunction that develops in TMEV mice (Figure 1B). ABX treatment induced dysbiosis, since the bacterial 16S gene was not amplified from fecal pellets of TMEV-ABX mice under standard PCR conditions (Figure 1C). However, in forced PCR conditions to amplify bacterial DNA from TMEV samples, the bacterial composition of the stools analyzed by Illumina MiSeq sequencing revealed that the microbiota clustered according to the treatment received (ABX or vehicle: Figure 1D). ABX treatment induced a decrease in the relative abundance of *Bacteroidetes* and *Firmicutes*, and an increase in *Actinobacteria* and *Proteobacteria* phyla (Figure 1E). At the genus level, ABX induced a decrease in the relative abundance of *Oscillospira, Ruminococcus* (phylum *Firmicutes*), *Anaeroplasma* (phylum *Tenericutes*) *Mucispirillum* (phylum *Deferribacteres*) and *Bilophila*, and *Sutterella* (phylum *Proteobacteria*), while there was an increase in *Anaerococcus, Streptococcus, Bacillus* (phylum *Firmicutes*), Flavobacterium, *Prevotella* (phylum *Bacteroidetes*), *Acinetobacter, Ochrobactrum* (phylum *Proteobacteria*), and *Corynebacterium* (phylum *Actinobacteria*: Figure 1F).

We used PICRUSTs to assess the functional content of the microbiota based on the 16S data. In TMEV mice that received ABX the abundance of several KEGG pathways was enhanced, specifically those related to Infectious Diseases, Cell motility and secretion, Neurodegenerative Diseases, Signal transduction, Cell Growth and Death, or Biosynthesis and biodegradation of secondary metabolites in treatment. By contrast, the KEGG pathways Endocrine System, Carbohydrate metabolism or Energy metabolism pathways were significantly dampened relative to the TMEV mice (Figure 1G).

### Oral Administration of Probiotics Modifies Microbiota Recolonization After Antibiotic Cessation, Influencing Deambulatory Activity, and Plasma Microbiota Metabolites in TMEV-IDD

**Figure 2:**
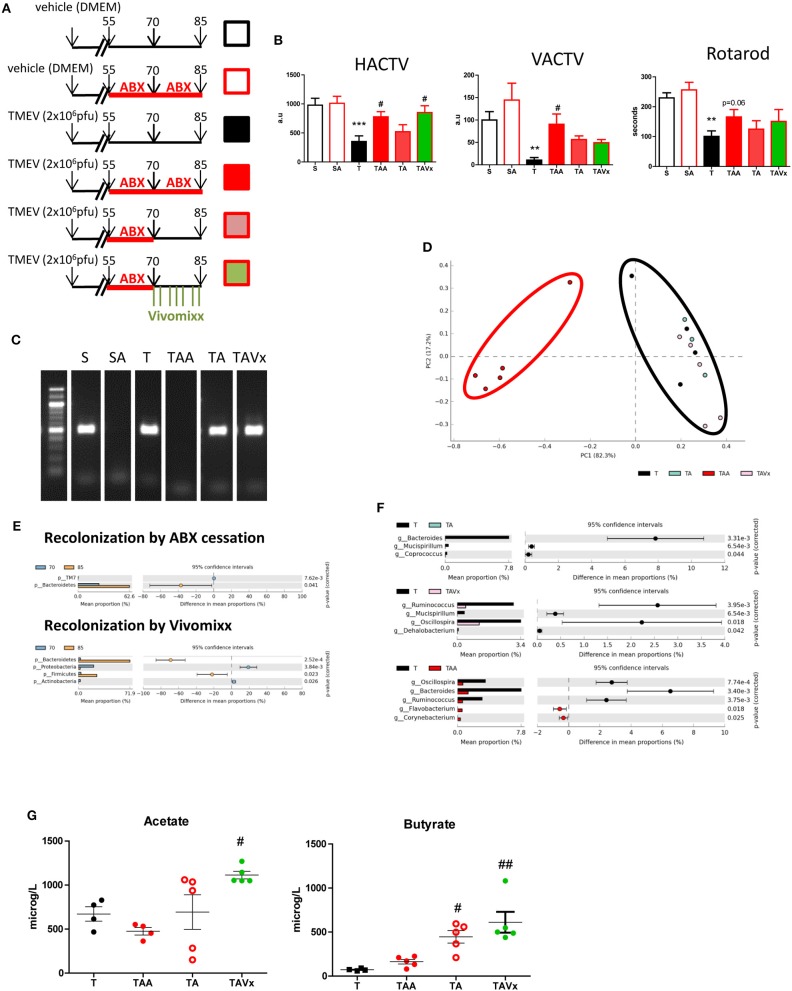
Partial recolonization after cessation of ABX aggravates motor disability in TMEV-IDD. **(A)** Scheme of the experimental groups: (S) black open squares, sham mice orally administered autoclaved water; (SA) red open square, Sham mice subjected to ABX treatment from day 55 to 85 pi; (T) black square, TMEV mice administered autoclaved water; (TAA) red square, TMEV mice administered ABX from day 55 to 85 pi; (TA) pink square, TMEV mice administered ABX from day 55 to 70 pi followed by autoclaved water; (TAVx) green square, TMEV mice administered ABX from day 55 to 70 pi followed by oral Vivomixx administration from 70 to 85 dpi. **(B)** Horizontal (HACTV) and vertical activity (VACTV) was evaluated for 10 min in the Activity cage, while motor coordination was measured for 5 min on the Rotarod apparatus. The data are presented as the means ± SEM: **p < 0.01 vs. S; ***p < 0.001 vs. S; ^#^*p* < 0.05 vs. T. **(C)** Agarose gel (1.5%) to visualize the amplicon amplified by 16S (V3–V4) PCR. **(D)** Principal components analysis of the faecal microbiota from the T (black; *n* = 4); TAA (red; *n* = 5); TA (blue; *n* = 3); TAVx (pink; *n* = 5) experimental groups. **(E)** Extended error bar plot identifying significant differences between the mean proportions of bacterial taxa upon recolonization following termination of the ABX treatment or recolonization upon Vivomixx administration. **(F)** Extended error bar plot to visualize significant differences in the bacterial taxa between the T and TAA group, or the TA or TAVx experimental groups. **(G)** Quantification of the plasma levels of acetate and butyrate. The data are presented as the means ± SEM: ^#^*p* < 0.05 vs. T; ^*##*^*p* < 0.01 vs. T.

In order to examine the effects of microbiota recolonization in the TMEV-IDD model, mice that received ABX until day 70 pi were divided into three groups: one that continued to receive ABX until day 85 pi (TAA); another that received vehicle alone (TA); and the third group that received the probiotic Vivomixx (TAVx: see Figure 2A). Motor deficits were more clearly evident in the group of TMEV mice that received the vehicle alone (Figure 2B), which displayed reduced deambulatory capacity (*p* < 0.001), less vertical activity (*p* < 0.01) and a shorter latency to fall in the rotarod test (*p* < 0.01). By contrast, oral ABX treatment dampened the motor symptomatology (as also seen at 70 dpi), evident through the horizontal (*p* < 0.05) and vertical activity (*p* < 0.05) in the activity cage, and while rotarod performance appeared to improve relative to the TMEV mice that received the vehicle alone, it did not reach statistical significance (*p* = 0.06). When ABX treatment was disrupted between 70 and 85 dpi, the motor behavior of the mice assessed in the activity cage and rotarod worsened (Figure 2B). Interestingly, the deambulation of mice that received the probiotic Vivomixx during the recolonization from day 70 to 85 pi improved (*p* < 0.05) although the probiotic failed to restore vertical activity that is the best indicator of hind limb weakness. Collectively these data show that microbiota removal by ABX affects the motor symptomatology being protective against TMEV-IDD, while recolonization disrupts this beneficial effect. Our results also suggest that the probiotic administration may help recolonization in a positive manner.

To corroborate the recolonization of the microbiota, fecal samples were collected at 85 dpi and analyzed in a similar manner to the samples from mice at day 70 pi. Amplification of 16S bacterial genes from fecal pellets showed that cessation of ABX followed by probiotic administration induced a rapid recolonization (Figure 2C). In addition, the analysis of the microbiota confirmed that samples from mice that no longer received ABX or that were administered probiotics cluster together with those that never received ABX (Figure 2D). As we could expect, the oral administration of Vivomixx significantly increased members of both the *Bacteroidetes* and *Firmicutes* phyla. However, it was noteworthy that ABX cessation mainly induced an increase in members of the phylum *Bacteroidetes* (Figure 2E). Despite this initiation of recolonization, the *Bacteroides* (phylum *Bacteroidetes*) *Mucispirillum* (phylum *Deferribactere*s), and *Coprococcus* (phylum *Firmicutes*) were relatively less abundant in the TA mice than in the TMEV mice (T), while there was significantly less *Ruminococcus, Dehalobacterium*, and *Oscillospora* (phylum *Firmicutes*) and *Mucispirillum* (phylum *Deferribacteres*) in the ABX-TMEV mice that received Vivomixx (TAVx) than in the TMEV mice. The continued treatment with ABX until day 85 maintained a decrease in the relative abundance of *Oscillospora* and *Ruminococcus* (phylum *Firmicutes*), and an increase in that of Flavobacterium (phylum Bacteroidetes) and *Corynebacterium* (phylum *Actinobacteria*) in the fecal pellets of TAA mice (Figure 2F).

There is evidence of the importance of gut microbiota derived fermentative metabolites like short chain fatty acids (SCFAs), such as acetate and butyrate, in autoimmunity and MS (38). Altered SCFA levels in blood could be associated to changes in gut microbiota so, we analyze the concentrations of acetate, and butyrate in plasma of mice subjected to the different treatments (Figure 2G). We observed a significant increase in the levels of acetate during recolonization in the mice that received Vivomixx relative to the TMEV mice (*p* < 0.05). The elevated levels of butyrate evident during the recolonization period in the absence of probiotics (*p* < 0.05 vs. TMEV mice) likely indicate that the adjustments to the microbiota populations after ABX cessation promote butyrate production, and the elevated plasma levels of this metabolite suggest that the oral administration of Vivomixx reinforces butyrate producing bacteria (*p* < 0.01 vs. TMEV mice).

### Gut Microbiota Recolonization Modifies the CNS Immunological Changes Provoked by Oral Antibiotic Treatment

**Figure 3:**
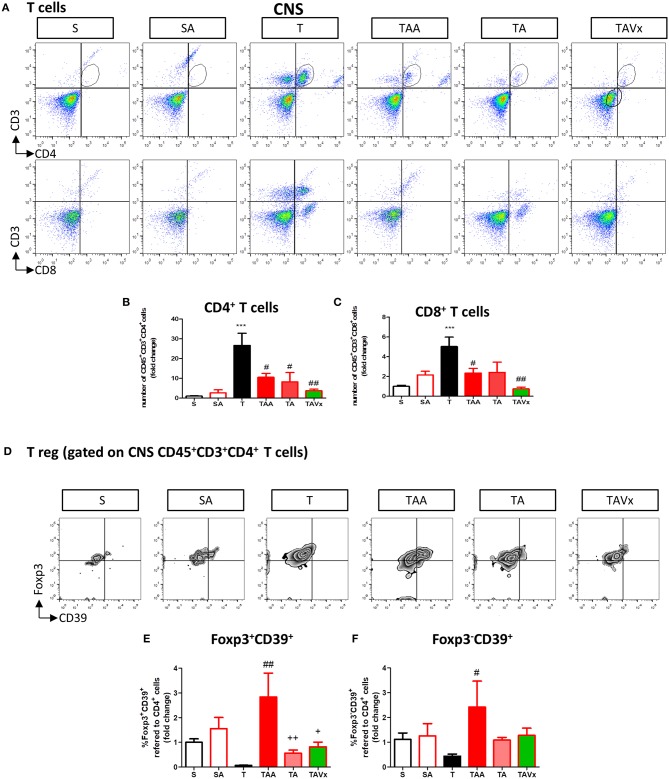
Changes in T cells in the CNS associated with both dysbiosis and recolonization of the gut microbiota. A single cell leukocyte suspension was obtained from the brain and spinal cord. **(A)** Representative flow cytometry plots of CD45^+^CD3^+^CD4^+^ T cells, CD45^+^CD3^+^CD8^+^ T cells. **(B,C)** Quantification of the change in number of CD45^+^CD3^+^CD4^+^ and CD45^+^CD3^+^CD8^+^ T cells relative to S mice. **(D)** Representative flow cytometry plots of CD39^+^Foxp3 cells gated on CD45^+^CD3^+^CD4^+^ T cells. **(E,F)** Percentage of Foxp3^+^CD39^+^ cells and Foxp3^−^CD39^+^ T cells gated on CD45^+^CD3^+^CD4^+^ T cells, respectively. The data represents the change relative to S mice. Groups S, T, and TAA (*n* = 10), and groups SA, TA, TAVx (*n* = 5). Statistics: ***p < 0.001 vs. S; ^#^*p* < 0.05 vs. T; ^*##*^*p* < 0.01 vs. T; ^+^*p* < 0.05 vs. TAA; ^++^*p* < 0.01 vs. TAA.

A flow cytometry study of the immune response revealed a significant increase in CD4^+^ T cells in the CNS of TMEV infected mice compared to the sham mice (*p* < 0.001), which is indicative of CNS infiltration during the chronic phase of TMEV-IDD (Figures 3A,B). In accordance with the data from EAE mice, oral ABX administration reduced the presence of CD4^+^ T cells relative to the TMEV mice (*p* < 0.05, Figure 3B), and this reduction in the number of CD4^+^ T cells persisted during the recolonization period (*p* < 0.05) and it was potentiated by the presence of Vivomixx (*p* < 0.01). A similar response was observed in the population of CD8^+^ T cells (Figures 3A,C), whereby an increase in the CD8^+^ T cells that infiltrated the CNS of TMEV mice (*p* < 0.001) was limited in the TMEV mice that were treated with ABX throughout the experiment (TAA group, Figures 3A,C). While there were fewer CD8^+^ T cells in the CNS of mice whose ABX treatment was withdrawn at 70 dpi (TA group), this was not significantly different to that observed in TMEV mice. However, there was a significant reduction in CD8^+^ T cells in the TAVX mice (*p* < 0.01) relative to the TMEV mice.

CD39 expressing CD4^+^ T cells are important modulators of autoimmune diseases like MS (39–42). Intriguingly, FACS analysis showed fewer Foxp3^+^CD39^+^ CD4^+^ Treg cells in the CNS of TMEV mice and while there was a significant increase after oral administration of ABX, this was reversed upon cessation of ABX treatment (Figures 3D,E). ABX treatment also augmented the subpopulation of Foxp3^−^CD39^+^ T cells in the CNS while no significant variations were found in this subpopulation under recolonization conditions (Figures 3D,F).

**Figure 4:**
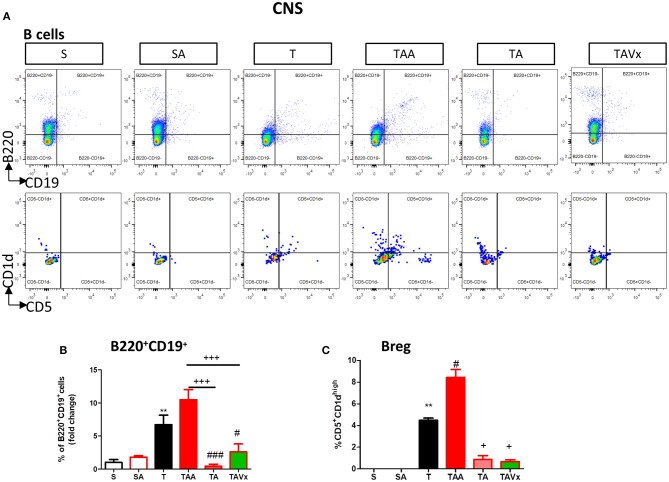
Changes in B cells in the CNS associated with both dysbiosis and recolonization of the gut microbiota. A single cell leukocyte suspension was obtained from the brain and spinal cord. **(A)** Representative flow cytometry plots of B220^+^CD19^+^ B cells and CD5^+^CD1d cells, gated on B220^+^CD19^+^ B cells. **(B)** Quantification of the change in the percentage of B220^+^CD19^+^ B cells relative to the S mice. **(C)** Percentage of CD5^+^CD1d^high^ cells in the CNS gated on B220^+^CD19^+^ B cells. Groups S, T and TAA (*n* = 10), and groups SA, TA, TAVx (*n* = 5). Statistics: **p < 0.01 vs. S; ^#^*p* < 0.05 vs. T; ^*###*^*p* < 0.001 vs. T; ^+^*p* < 0.05 vs. TAA; ^+++^*p* < 0.001 vs. TAA.

The experimental evidence obtained in the EAE model suggests that distinct subpopulations of B cells with regulatory phenotypes are involved in protection from the disease (43). Interestingly, there was an increase in the percentage of B cells (B220^+^CD19^+^) in the CNS of TMEV mice during the chronic phase when compared to the sham conditions (*p* < 0.01, Figures 4A,B). CNS of mice treated with ABX show similar percentage of B than TMEV mice, whereas cessation of ABX (*p* < 0.001) as well as ABX withdrawn plus the administration of Vivomixx (*p* < 0.05) provoked a significant reduction in B cells. One interesting observation (Figures 4A,C) was that the percentage of regulatory B cells (CD5^+^CD1^high)^ increased in TMEV mice (*p* < 0.01) and that the ABX cocktail enhanced this population of CNS regulatory B cells (*p* < 0.05) relative to that found in TMEV mice. In the rest of the conditions there were very few or none of these specific Breg cells. Collectively, our results reflect the importance of changes in the microbiota for CNS immunity in the context of the chronic neurodegenerative stage of TMEV-IDD.

### Microglial Changes and Axonal Damage Associated With Alterations in the Microbiota During the Chronic Phase of TMEV-IDD

**Figure 5:**
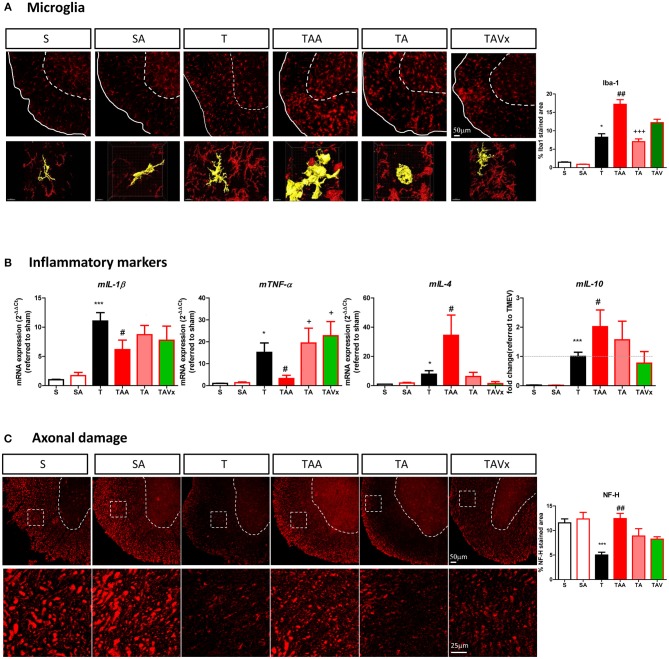
Dysbiosis and the recolonization of the gut microbiota are associated with changes to microglia and axonal damage in the spinal cord. **(A)** Representative images of transversal spinal cord sections immunostained with Iba-1 to label microglia. Scale bar 50 μm. In the bottom it is represented higher magnification reconstruction images (63x) of Iba1 stained cells to show the changes in morphology. Scale bar 10 μm. These images are accompanied by a quantification of the relative area occupied by Iba-1 staining. **(B)** Expression of the inflammatory markers (*mIL-1b, mTNF-*α, *mIL-4*, or *mIL-10*) in the spinal cord was assessed by qRT-PCR analysis and using the 2^−ΔΔCt^ method. **(C)** Representative images of axonal damage in the spinal cord visualized by Neurofilament-H staining (Scale bar 50 μm). Higher magnification pictures are also presented (Scale bar 25 μm) along with quantification of the area stained with Neurofilament-H. Statistics: *p < 0.05 vs. S; ***p < 0.001 vs. S; ^#^*p* < 0.05 vs. T; ^*##*^*p* < 0.01 vs. T; ^+^*p* < 0.05 vs. TAA; ^+++^*p* < 0.01 vs. TAA.

As innate immune cells, microglia are critical to maintain CNS homeostasis and interestingly, the microbiota may shape microglial activity (44–46). Here we found a greater area of Iba-1 microglial staining in the spinal cord of TMEV mice during the chronic phase relative to that in sham mice (*p* < 0.05, Figure 5A), consistent with our previous data (30). Nevertheless, microglia undergo morphological changes when confronted by different microbiota challenges (Figure 5A). For example, cells adopt a transitional morphology in TMEV mice that received ABX, shifting from cells with thin cell bodies with numerous branched extensions to more round amoeboid-like cells with fewer branches and more thickened then, the area of Iba-1 staining increased relative to that in TMEV mice (*p* < 0.01). During recolonization, the microglia maintained their amoeboid shape but the area of Iba-1 staining reduced significantly relative to the ABX-TMEV mice (*p* < 0.001). By contrast, the mice that received oral Vivomixx had microglia with more branches than those observed during recolonization in the absence of probiotics. 3D reconstruction videos of representative microglial cells to visualize the morphological changes of microglia from TMEV and ABX-TMEV mice are provided as Supplementary Material (T, Supplementary Video 1; TAA, Supplementary Video 2).

The expression of the genes encoding the pro-inflammatory cytokines m*IL-1*β and m*TNF-*α was assessed in the spinal cord of mice subjected to the different conditions studied, together with those encoding the anti-inflammatory cytokines m*IL-4* and m*IL-10* (Figure 5B). Treatment with ABX diminished the levels of *mIL-1*β and m*TNF-*α induced by TMEV, and the recolonization after cessation of ABX and probiotic Vivomixx administration reversed this effect in the case of m*TNF-*α. Regarding the anti-inflammatory cytokines, oral ABX treatment significantly increased m*IL-4* and m*IL-10* mRNA expression relative to TMEV mice (*p* < 0.05), these genes being expressed more strongly than in sham mice (*p* < 0.001). After cessation of ABX administration there was a tendency for *mIL-4* mRNA expression to decrease, whereas Vivomixx treatment reversed IL-4 gene expression to a similar level as in sham mice. However, the changes in *mIL-10* gene expression were more subtle during such recolonization. A histological analysis of spinal cord sections stained for NF-H was performed to quantify the axonal integrity. The axonal damage in TMEV mice was prevented by ABX treatment (*p* < 0.01) and recolonization tended to limit this effect, inasmuch as the damage was not significantly different to that in TMEV mice (Figure 5C).

### Peripheral Immunological Changes in TMEV-IDD Mice Associated With Oral Antibiotic Treatment and Recolonization With or Without Probiotics

**Figure 6:**
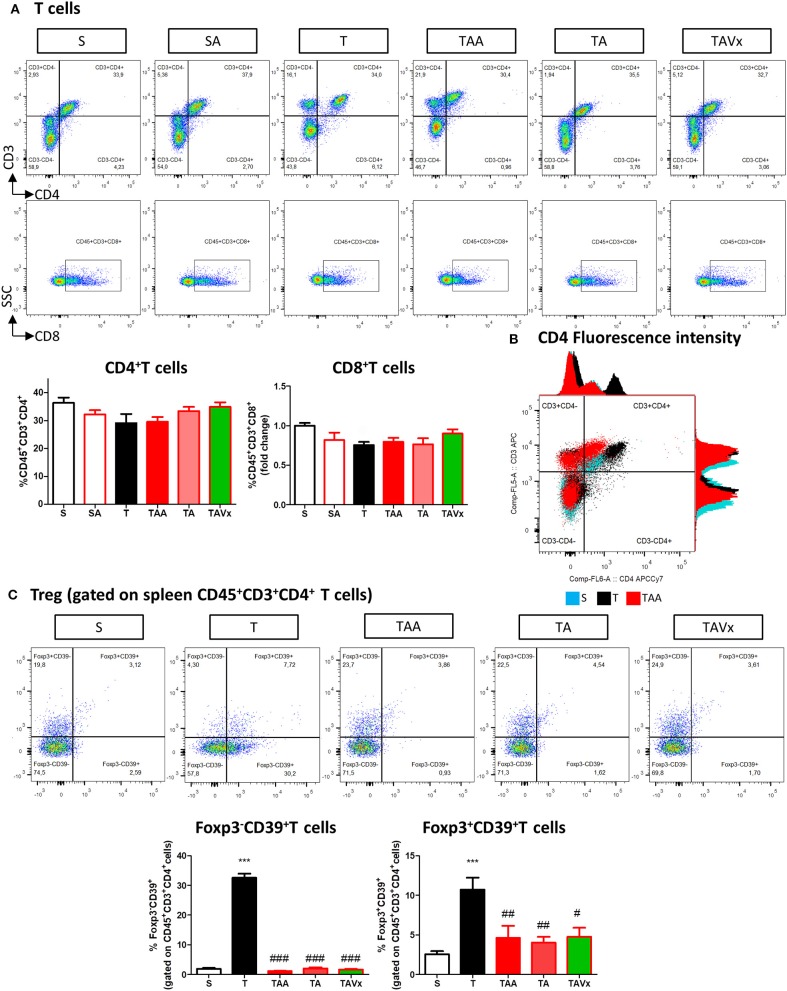
Gut microbiota manipulation effect on the spleen T cells in mice. A spleen cell suspension was stained to analyse CD4^+^, CD8^+^ T cell, and CD39^+^ T cell **(A)** Representative flow cytometry plots of CD45^+^CD3^+^CD4^+^ T cells and CD45^+^CD3^+^CD8^+^ T cells are presented along with the corresponding quantification below. **(B)** Histogram of CD3 and CD4 fluorescence intensity emitted by CD45^+^CD3^+^CD4^+^ splenocytes from representative mice of S, T, and TAA experimental groups. The arrow marks the increase of CD4 fluorescence intensity by splenocytes from TMEV mice **(C)** Flow cytometry plots of Foxp3CD39^+^ T cells gated on CD45^+^CD3^+^CD4^+^ T cells shown together with the quantification of the Foxp3^−^CD39^+^ and Foxp3^+^CD39^+^ T cells. Statistics: ***p < 0.001 vs. S; ^#^*p* < 0.05 vs. T; ^*##*^*p* < 0.01 vs. T; ^*###*^*p* < 0.001 vs. T.

**Figure 7:**
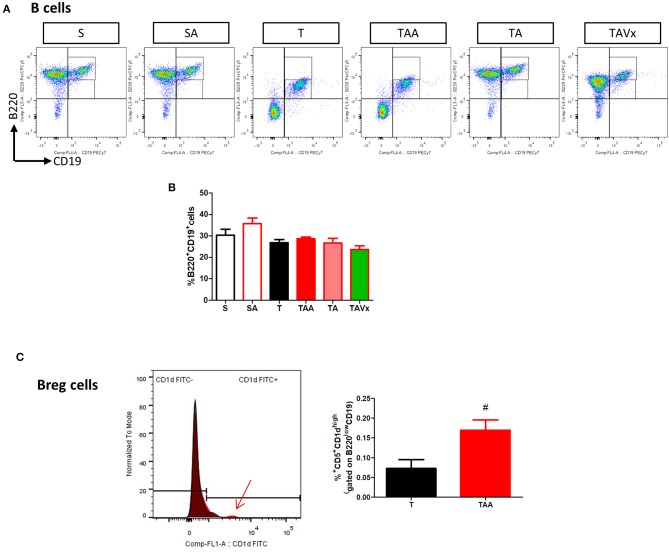
Gut microbiota manipulation effect on the spleen B cells in TMEV mice. **(A)** Representative flow cytometry plots of percentage of B220^+^CD19^+^ B cells are presented along with the corresponding quantification below **(B)**. **(C)** Normalized histogram of CD1d expression in B220^low^CD19^+^CD5^+^ cells is presented along with the quantification of the percentage of B220^low^CD19^+^CD5^+^CD1d^high^ Breg cells. Statistics: ^#^*p* < 0.05 vs. T.

Minimal changes were detected in the populations of CD4^+^ and CD8^+^ T cells in the spleen of the TMEV-mice (Figure 6A). However, it was noteworthy that CD4 fluorescence intensity was higher in TMEV mice than in sham, fact that was prevented by ABX (Figure 6B). In addition, the proportion of FoxP3^−^CD39^+^CD4^+^ cells were more prevalent in the TMEV mice than in the sham mice (*p* < 0.001), whereas oral ABX administration significantly reduced the proportion of these cells relative to the TMEV mice (*p* < 0.01), contrasting with the data from the CNS. A similar profile was observed for the subpopulation FoxP3^+^CD39^+^CD4^+^ cells, which was enhanced in the TMEV mice (*p* < 0.001 vs. Sham), and these cells were less prevalent in the spleen of mice that received oral ABX (*p* < 0.001 vs. TMEV). Both, recolonization alone or in conjunction with Vivomixx did not modify the reduction in the Foxp3^+^CD39^+^CD4^+^ and Foxp3^−^CD39^+^CD4^+^ T cell subpopulations provoked by ABX (Figure 6C). Again, in contrast to the CNS, we did not observe changes in the frequency of B cells under the distinct conditions used (Figures 7A,B). However, when we gated for B220^low^CD19^+^CD5^+^ there was a small, yet significant, increase in CD1d^high+^ cells in ABX TMEV mice relative to the TMEV mice (*p* < 0.05, Figure 7C). Therefore, the manipulation of the intestinal microbiota affected differentially the central and peripheral (spleen) immune responses in the context of neurodegenerative inflammation.

**Figure 8:**
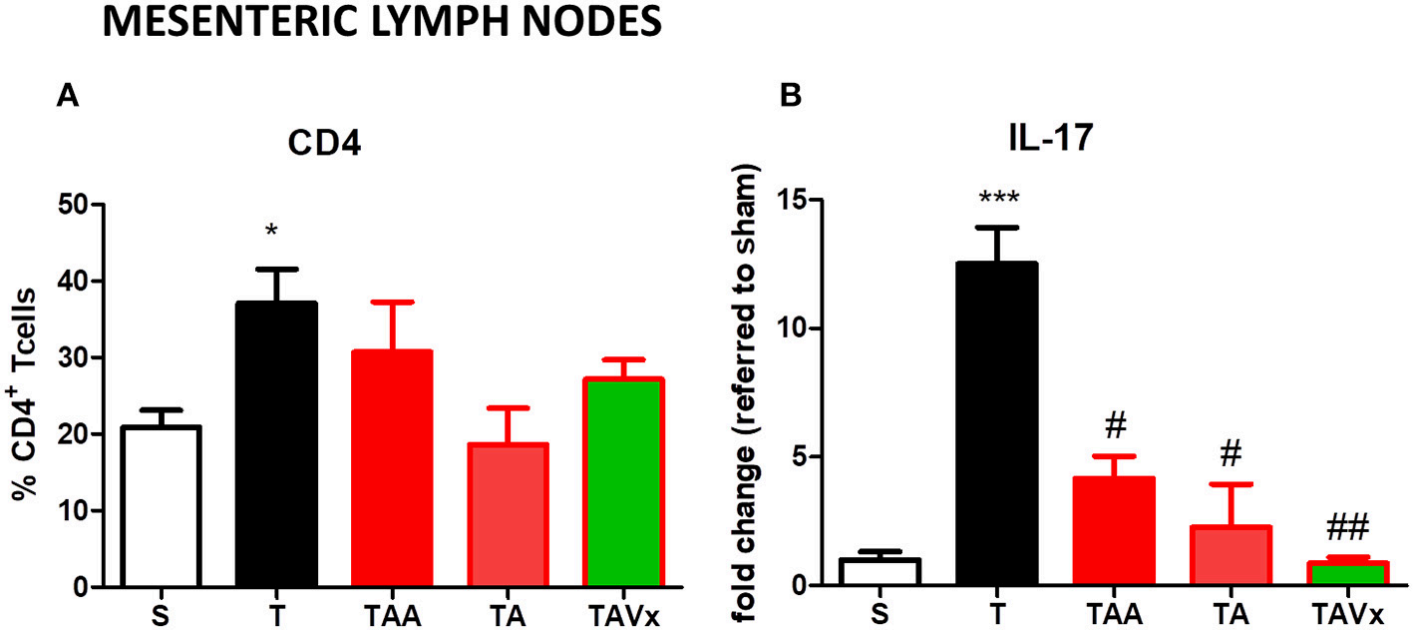
Manipulation of the gut microbiota affects the Th17 responses in the MLN. **(A)** The frequency of CD4^+^T cells increased in TMEV mice relative to sham mice. **(B)** CD4^+^ T cells isolated using Miltenyi beads were polarized to Th17 in culture, and IL-17 production was measured by ELISA. The data represent the mean ± SEM of the change relative to Sham (data from two experiments performed in triplicate). Statistics: *p < 0.05 vs. S. ***p < 0.001 vs. S; ^#^*p* < 0.05 vs. T; ^*##*^*p* < 0.01 vs. T.

TMEV-infected mice show a significant increase (*p* < 0.05 vs. sham mice) in the proportion of CD4^+^T cells in MLNs, but no significant changes were observed in this population under the different conditions of gut microbiota manipulation (Figure 8A). The cytokine IL-17 was quantified by ELISA in CD4^+^ lymphocytes harvested from the MLNs of mice after their polarization to the Th-17 phenotype. CD4^+^T cells obtained from TMEV mice produced significantly more IL-17 (*p* < 0.001) than those from sham mice, whereas oral treatment with ABX reduced the production of IL-17 in TMEV mice (*p* < 0.05) (Figure 8B). Strikingly, there was significantly less IL-17 after ABX cessation relative to TMEV mice and the presence of Vivomixx potentiated this reduction (*p* < 0.01; Figure 8B).

## Discussion

In the last 10 years, evidence has emerged of a relationship between gut microbiota and MS not only in animal models but also in patients (13, 14, 47–49). However, the influence of the gut microbiota on progressive forms of MS is an issue little explored. Hence, in a progressive model of MS we investigated how microbiota manipulation influences the central and peripheral immune responses, axonal preservation integrity and motor function as an index of disease progression.

During the chronic phase of TMEV-IDD, once the symptomatology had been established, changes in the composition of the microbiota were evident between sham and TMEV mice (30). In the present study the oral ABX cocktail administered during the pre-symptomatic transitional phase prevented motor dysfunction of TMEV mice. The analysis of the bacterial composition in feces revealed a reduction of the relative abundance of bacteria belonging to *Firmicutes* and *Bacteroidetes* phyla, while increasing bacteria belonging to *Actinobacteria* and *Proteobacteria*. Accordingly, previous studies on EAE-mice treated with the same ABX cocktail indicated a decrease of Firmicutes abundance (50) or a macroscopic GF-like phenotype (51). Particularly, vancomycin reduced the abundance of *Firmicutes* and *Bacteroidetes* phyla as well as increased *Proteobacteria* abundance (52). Although an increase of relative abundance of *Proteobacteri*a has been related to both, pro and anti-inflammatory roles in MS (53), our observations and the data published in the EAE model suggest a beneficial role of this commensal population. It must be note that variations on microbiota composition depend on the antibiotic cocktail, doses, timing of treatment, or even mice strain.

The association of the different bacterial communities to the gut-brain-immune system interaction is currently under study. It is therefore significant that *Prevotella* abundance is less prominent in MS patients (13, 14) and its presence is enhanced by disease-modifying therapy (14). Moreover, using a human leukocyte antigen (HLA) class II transgenic mouse model of EAE, increasing human gut-derived commensal *Prevotella* was able to suppress the disease (54). In our study the relative abundance of *Prevotella* was enhanced in TMEV-mice treated with ABX that displayed a good performance in the motor tests, supporting the possible therapeutic role of *Prevotella* in MS.

Recolonization following the cessation of ABX had a negative effect on TMEV-IDD progression, as reflected by the worsened motor function and in accordance to previous studies on GF mice after directed intestinal colonization with segmented filamentous bacteria (20). Indeed, there is significant support for the gut microbiota playing a critical role in shaping the immune system (55–58). Here, we provide evidence of marked differences between central and peripheral immune responses in mice in which the microbiota had been manipulated. Interestingly, oral ABX limits the increase in the number of CD4^+^ and CD8^+^ T cells in the CNS of TMEV mice, which unexpectedly persisted in TMEV mice when the ABX treatment stopped. However, analyzing the subpopulations of CD4^+^ T cells in more depth, recolonization reversed the increase in both Foxp3^+^CD39^+^ T cells and Foxp3^−^CD39^+^ T cells induced by ABX treatment in TMEV infected mice, supporting the beneficial role of CD39^+^ Treg cells in preventing MS progression. In fact, one of the pathways by which the commensal microbiome affects host immunity at the distal site is by regulating the activity of Treg cells through the ectonucleotidase CD39 (59). Particularly, Foxp3^+^CD39^+^ T cells isolated from MS patients supress IL-17 production and there was a significant reduction in the CD39^+^ Treg cells in MS patients (39). CD39^+^ cells have been associated with the suppression of neuroinflammation by *Bacteroides fragilis* in the EAE model (60) in accordance with studies showing that polysaccharide A (PSA) was not able to protect against EAE in CD39^−^deficent mice (59). Moreover, CD39^+^ Treg cells are thought to be involved in the beneficial effects of Teriflunomide in the EAE model (40) and an increase in CD39 expressing cells occurs in MS patients that receive Fingolimod (61). It is well-known that CD39 Treg cells play an essential role in constraining pathogenic Th17 cells and preventing autoimmune disease, potentially involving both the IL-10 and TGF-β produced by Foxp3^+^CD39^+^ and Foxp3^−^CD39^+^ cells, respectively (62).

Although MS has classically been considered a predominantly T cell-dependent autoimmune disease, B cells also regulate immune responses and contribute to disease pathogenesis (63, 64). In particular, regulatory CD5^+^CD1^high^ B cells that produce IL-10 were recently shown to impede EAE initiation (65), and broad spectrum antibiotic administration to EAE mice increased the CD5^+^CD1^high^ B cell population at distal lymphoid sites (43). Here, ABX treatment was seen to augment the regulatory CD5^+^CD1d^high^ B cells in the spleen and CNS, which could be partially responsible for the increase in IL-10 in the spinal cord of TMEV-ABX mice. Indeed, other leukocyte populations, including T lymphocytes, monocytes, and intrinsic CNS microglia might be contributing to the increased gene expression of IL-10. Hence, the findings presented here suggest that gut commensal bacteria might mediate CNS immune homeostasis.

The main changes referred to gut microbiota manipulation in the TMEV-IDD model were in the CNS where microglia are the baseline sentinels. The alterations in microglial morphology induced by oral ABX are indicative of the dynamic polarization states of these cells. For instance, microglia can retract their processes and enlarge their cell body like activated phagocytic cells, increasing the relative area of Iba-1 staining. As commented before, the anti-inflammatory gene expression profile in the spinal cord was associated with reduced *IL-1*β, and increased *IL-4* and *IL-10* mRNA expression. Classically, microglial activity is influenced by cytokines, chemokines and other micro-environmental factors, shaping their homeostatic and pathogenic program (66). Recent studies show striking differences in the microglia derived from GF and specific pathogen free (SPF) mice at the genetic and morphological level (44), and signals from maternal gut microbes could influence microglia even at birth (67). We suggest that the changes in the microbiota produced by oral ABX maintain microglia in a protective state, attenuating the damage associated with chronic neuroinflammation that occurs in progressive MS. Ameboid microglial cells were also present in the spinal cord during recolonization. However, the Iba-1 stained area was significantly smaller than that observed in TMEV mice that received ABX, likely suggesting a reduction in the protective microglia recruited to inflammatory sites. More ramified microglia was associated to Vivomixx treatment, although the spinal cord gene expression profile during recolonization was pro-inflammatory. Most importantly in this context, is that oral ABX administration preserves the axonal integrity in the spinal cord of TMEV mice while recolonization increased the axonal damage.

To shed further light on our data, we measured in plasma the SCFAs, acetate and butyrate (68). Remarkably, the levels of acetate and butyrate in the plasma only increased during recolonization, particularly after the application of Vivomixx, and probably, in association with an increase of *Bacteroidetes* and *Firmicutes* in the feces. Therefore, plasma acetate and butyrate changes seem to reflect critical events in the recolonization period after long term oral ABX administration, suggesting the interest of using probiotics to help recolonization. Although SCFAs ameliorate the disease course of EAE (68, 69), few studies have addressed the role of these metabolites in MS patients. While MS patients might exhibit a reduction in SCFA producing bacteria (70), in our study only mice that received Vivomixx during recolonization improved their deambulation. Whether the enhanced deambulatory activity is related to an increase in acetate and butyrate remains unclear, an issue that merits further study. Our findings also indicate that alterations to bacterial populations during recolonization induce changes in CNS immunity, being the most significant the diminished CD39^+^ Treg and Breg cells following microbiota depletion by oral ABX.

Unlike EAE, the main changes provoked by manipulating the gut microbiota in the TMEV-IDD model were evident at the CNS with modest changes in the periphery. Although EAE and TMEV-IDD share pathogenic mechanisms, these two models have important differences that may reflect mouse strain (C57BL/6 vs. SJL/J) specific responses regarding immunity, neuroinflammation and clinical outcomes. This would explain why our main findings after gut microbiota manipulation occur in the CNS while the alterations in peripheral compartments are mild in comparison with the changes observed in EAE in the same context. At spleen level, ABX inhibited the induction of CD4 expression observed in the CD4^+^ T cells from TMEV mice; this fact is relevant since these CD4^high^ cells, and not CD4^norm^ cells, cause EAE after adoptive transfer to naïve recipients (71). The contribution of the MLN to pro-inflammatory Th-17-cell generation during inflammation of the small intestine has been reported previously (72) and dendritic cells exposed to PSA migrate to the MLN, where they favor the presence of IL-10 producing Treg cells regulating the Th1/Th17 response in Crohn's disease (73). Our *ex vivo* experiments with sorted CD4^+^ T cells from MLNs cultured in conditions favoring Th-17 polarization generated interesting data. Firstly, CD4^+^ T cells from TMEV mice were capable of further enhancing IL-17 production and secondly, CD4^+^ cells from ABX-TMEV mice released less IL-17, consistent with previous studies (18). However, recolonization failed to reverse the reduction in IL-17 and Vivomixx was not sufficient to recover the ABX-induced suppression of IL-17 by CD4^+^ T cells sorted from the MLN.

One of the main messages of our study is precisely that at the chronic neurodegenerative phase of TMEV-IDD, the main immunological changes related to the manipulation of the gut microbiota occur within the CNS. Our data also support a link gut microbiota-CNS axis, since microbiota dysbiosis provoked by oral ABX exerts neuroprotective effects, diminishing motor disability and axonal damage. Nevertheless, exactly how the gut microbiota modulates the development and disease course of TMEV-IDD is still unclear. Further research is necessary to better understand the mechanisms and signaling pathways involved in coordinating the gut microbiota and the CNS in progressive forms of MS.

## Data Availability

The raw data supporting the conclusions of this manuscript will be made available by the authors, without undue reservation, to any qualified researcher.

## Ethics Statement

All experiments were performed in strict accordance with EU (Directive 2010/63/EU) and Spanish regulations (Royal Decree 53/2013 BOE n° 34 and Comunidad de Madrid decree: ES 280790000184). The Ethics Committee on Animal Experimentation at the Instituto Cajal (CSIC) also approved all the procedures employed in this study (protocol number: 2013/03 CEEA-IC).

## Author Contributions

LM and CG designed the research. LM, FC-S, MM, and AF performed the research. CE, JÁ-C, and LV were advisors involved in the discussion of the results. LM and CG wrote the manuscript. All the authors contributed to and approved the final version of the manuscript submitted for publication.

### Conflict of Interest Statement

The authors declare that the research was conducted in the absence of any commercial or financial relationships that could be construed as a potential conflict of interest.

## Supplementary Material

Supplementary Video 1 3D reconstruction of microglia cells from TMEV-mice. Digital reconstruction by IMARIS software of microglial cells surface obtained from 63x confocal image from the spinal cord of TMEV infected mice. Red cells correspond to Iba-1 positive cells. The morphology of representative aisled cell are presented in yellow and DAPI in blue.

Supplementary Video 2 3D reconstruction of microglia cells from ABX-TMEV mice. 63x confocal image from transversal spinal cord section from TMEV mice subjected to ABX treatment were processed by IMARIS software and 3D reconstruction of microglial cells were obtained. Red cells correspond to Iba-1 positive cells. The morphology of representative cells is showed in yellow and an isolated cell could not be dis distinguished. DAPI staining for nucleus is present in blue.
