# Expression of Concern: Manipulation of Gut Microbiota Influences Immune Responses, Axon Preservation, and Motor Disability in a Model of Progressive Multiple Sclerosis

**DOI:** 10.3389/fimmu.2020.614941

**Published:** 2020-10-27

**Authors:** Frontiers Editorial Office

**Affiliations:** Frontiers Media SA, Lausanne, Switzerland

**Keywords:** Theiler’s virus model, gut microbiota, Treg and Breg cells, neuroinflammation, multiple sclerosis

The publisher has been alerted to potential errors in the stated probiotic composition of the product used in this study. This statement will remain while an investigation is underway and will be updated and any necessary corrections made at the conclusion of the investigation.

